# Utilization of Sustainable Ingredients (Cañihua Flour, Whey, and Potato Starch) in Gluten-Free Cookie Development: Analysis of Technological and Sensorial Attributes

**DOI:** 10.3390/foods13101491

**Published:** 2024-05-11

**Authors:** Olivia M. Luque-Vilca, Jover Y. Paredes-Erquinigo, Lenin Quille-Quille, Tania J. Choque-Rivera, Domingo J. Cabel-Moscoso, Thalía A. Rivera-Ashqui, Reynaldo J. Silva-Paz

**Affiliations:** 1 Facultad de Ingeniería de Procesos Industriales, Universidad Nacional de Juliaca, Av. Nueva Zelandia 631, Juliaca 21101, Peru; oluque@unaj.edu.pe (O.M.L.-V.); jover2022yoker@gmail.com (J.Y.P.-E.); l.quille@unaj.edu.pe (L.Q.-Q.); tj.choquer@unaj.edu.pe (T.J.C.-R.); 2 Ingeniería Ambiental, Universidad Nacional San Luis Gonzaga, Ica 11001, Peru; jesus.cabel@unica.edu.pe; 3 Facultad de Ingeniería y Arquitectura, Universidad Peruana Unión, km 19 Carretera Central, Ñaña, Lima 15457, Peru; thaliarivera@upeu.edu.pe; 4 Escuela de Ingeniería en Industrias Alimentarias, Departamento de Ingeniería, Universidad Nacional de Barranca, Av. Toribio de Luzuriaga N° 376 Mz J. Urb. La Florida, Barranca 15169, Peru

**Keywords:** Andean grain, whey, sensory, cookies, celiac

## Abstract

In recent years, the consumption of gluten-free products has increased due to the increasing prevalence of celiac disease and the increased preference for gluten-free diets. This study aimed to make cookies using a mixture of cañihua flour, whey, and potato starch. The use of a Box–Behnken design allowed for flexible ingredient proportions and physicochemical properties, centesimal composition, color, texture, and sensory attributes to be evaluated through consumer tests (Sorting and acceptability). The results highlighted significant variations in physicochemical data, composition, color, and texture across formulations. The blend with 38.51% cañihua flour, 10.91% sweet whey, 25.69% potato starch, 8.34% margarine, 11.10% sugar, 0.19% sodium chloride, 0.51% baking powder, 0.51% vanilla essence, and 4.24% egg exhibited superior sensory appeal. This formulation boasted excellent texture, aroma, flavor, color, and appearance, indicating high sensory and physicochemical quality. The use of cañihua flour, sweet whey, and potato starch not only provides a gluten-free option but also delivers a nutritious and sensorily pleasing choice for those with dietary restrictions. Future research could explore the commercial viability of producing these cookies on a larger scale, as well as investigating the potential health benefits of these ingredients.

## 1. Introduction

The rapid rise in the consumption of gluten-free products [1] can be attributed to the escalating prevalence of celiac disease globally (>1.30%) [2], a condition often undiagnosed despite evident symptoms [3]. Celiac disease, a chronic inflammatory bowel disorder affecting approximately one in every hundred individuals worldwide [4]. Peru has a prevalence of 1.20% in an age range between 18 and 29 years [5], was necessitates a strict gluten-free diet as the sole treatment option. Consequently, a heightened awareness of celiac disease [6] and non-celiac gluten sensitivity has fueled the demand for gluten-free products [7]. However, many available gluten-free products exhibit subpar nutritional profiles, often laden with excessive fats and sugars compared to their gluten-containing counterparts [8]. Thus, there is a pressing need to develop nutritionally complete, healthy, and palatable gluten-free alternatives that cater to consumer preferences [9].

The use of sustainable ingredients in food product development reflects a comprehensive commitment to long-term human, environmental, economic, and social welfare. Cañihua flour, whey, and potato starch are chosen for their natural and sustainable origins, including cañihua, a traditional Andean crop cultivated sustainably for centuries. Whey, a cheese production by-product, and potato starch, sourced from renewable outlets, emphasize resource efficiency and waste reduction. These ingredients also offer potential economic and social benefits by supporting local communities and promoting agricultural diversification, contributing to sustainable development.

In contemporary baking practices, there is a notable inclination towards utilizing high Andean crops [10], indigenous to the South American Andean regions and cultivated for centuries [11]. Among these, quinoa [12], amaranth [13], and cañihua [14], stand out as prime candidates due to their richness in essential amino acids and gluten-free nature, rendering them a wholesome and nutritious substitute [15] for conventional grains in baked goods. Cañihua, in particular, emerges as a promising contender owing to its protein content (ranging between 15 and 19%) [16] and a balanced amino acid profile, meeting the FAO/WHO/UNU recommendations [17].

Notably, cañihua boasts a plethora of bioactive compounds including phenolic acids (16.8 to 59.7 mg/100 g), soluble phenolic acids (7 to 61%), and flavonoids [18], endowing it with potential health benefits and therapeutic implications attributed to its antioxidant and anti-inflammatory properties [19]. Furthermore, cañihua is a rich source of essential minerals such as iron, calcium, magnesium, and zinc, as well as B complex vitamins including thiamine (B_1_), riboflavin (B_2_), niacin (B_3_), and folic acid (B_9_) [20].

However, the development of products like gluten-free cookies continues to pose technological challenges, particularly concerning texture and appearance [21]. Addressing these challenges necessitates the strategic incorporation of additives such as cross-linking enzymes, hydrocolloids, and amylose to enhance volume and stabilize the dough [22], thereby reducing hardness [23]. Additionally, the inclusion of native or modified starch enhances water retention, leading to increased volume and a softer texture [24], while the incorporation of whey improves the crust and provides both nutritional enrichment and sensory enhancement [25,26,27].

This emphasis on enhancing sensory attributes is pivotal, as taste and aroma significantly influence consumer acceptance [28]. Given the direct impact of inputs on the color and sensory attributes of cookies, the thorough evaluation of ingredients is imperative to optimize the overall product quality [29]. Experimental designs, such as the Box–Behnken design, offer a systematic approach to achieve this goal, facilitating the identification of optimal ingredient combinations and processes while reducing formulation time and costs [30,31].

Currently, various formulations tailored for individuals with gluten-related disorders have been developed, incorporating gluten-free inputs such as heat-treated cormorant Colocasia spp. flour, rice flour, rice proteins, peas, egg white, whey, β-conglycinin concentrate extracted from defatted soybean flour, and composite rice and chickpea flour [32,33,34,35]. These formulations underscore ongoing efforts to meet the dietary needs of individuals with gluten-related disorders, highlighting the versatility and potential of alternative ingredients in cookie production.

In this context, the present study aims to develop gluten-free cookies based on cañihua flour (*Chenopodium pallidicaule* Aellen), whey, and potato starch (*Solanum tuberosum*) while evaluating their physicochemical and sensory properties.

## 2. Materials and Methods

### 2.1. Raw Material

To make the cookies, cañihua flour (*Chenopodium pallidicaule* Aellen) variety INIA ILLPA was used, which was acquired from the National Institute of Agrarian Innovation (INIA)—Puno, ground in a disc mill and sifted with a mesh system N°. 40 particle size (0.18 mm). The native starch was obtained from the native potato variety Imilla negra acquired from INIA—Puno, using the wet milling technique with a slight modification [36]. The sweet whey was obtained from the pariah cheese making process. Margarine (Famossa), brown sugar (Cartavio), iodized kitchen salt (Emsal), baking powder and vanilla essence (Fleischmann), and eggs (Calera) were purchased from the supermarket in the city of Juliaca, Puno.

### 2.2. Methodology

#### 2.2.1. Preparation of the Cookie

The sweet cookie formulation from the American Association of Cereal Chemists [37] was used with modifications. Table 1 shows the percentages of the different formulations to be prepared for the research. The formulation includes the variation of cañihua flour, whey, and potato starch. The rest of the ingredients were margarine, sugar, sodium chloride, baking powder, vanilla essence, and liquid egg. The preparation process consisted of weighing the ingredients and mixing them in a dough mixer (Nova brand, model K25, Lima, Peru) to cream for 10 min at a level 2 speed; then, it was kneaded for 10 min at speed level 1 to continue with the lamination (Nova Brand, model MK500, Lima, Peru) until a thickness of 0.5 cm was reached. The samples were cut manually into circular pieces of 20 mm diameter with a mold. Finally, the baking process was carried out in a rotary oven (Nova Brand, Model MAX 1000, Lima, Peru) at 150 °C for 15 min [37]. The samples were evaluated after 24 h of storage at room temperature.

#### 2.2.2. Physicochemical Analysis and Proximal Composition

The physicochemical analysis was carried out on the cañihua flour, potato starch and whey. The humidity was quantified by the gravimetric method No. 950.46 and ashes by the method No. 935.08 [38], the pH by the NTP 206.014:1981 [39], the titratable acidity by the method described NTP. 206.013:2011 [40], and water activity (Aw) using an AquaLab vapor absorption analyzer (model VSA, Washington, DC, USA) [41]. In addition, the physicochemical properties and proximal composition of the cookies were then analyzed according to RM N°1020-2010/MINSA Sanitary Standard for the Manufacture, Preparation and Marketing of Baking, Cookies and Pastry Products [42] and the guidelines of the Association of Official Analytical Chemists—AOAC [38]: Moisture was calculated by the gravimetric method No. 950.46, proteins by the Kjeldahl method No. 984.13, fat by the Soxhlet method No. 203.05, crude fiber by method 962.09, ash by the method No. 935.08 and carbohydrates by difference. The energy value was calculated using: Energy value = 4(%carbohydrates) + 4(%Protein) + 9(%fat) expressed in Kcal/100 g [43].

#### 2.2.3. Colorimetric Properties

The color measurements, by method (CIE L*a*b*), of the cookies were obtained with a Konica Minolta colorimeter (model 700d, Ramsey, NJ, USA), where L* is defined as the Luminosity, a* as the red/green coordinates, and b* as the yellow/blue coordinates [44]. Each test was performed in triplicate. Additionally, the color intensity (chroma) was calculated: C* = root (a*^2^ + b*^2^) and the hue angle: h* = arctan (b*/a*).

#### 2.2.4. Instrumental Texture Analysis

Texture profile analysis was performed with the Instron universal testing equipment (model 34TM-5 Dual Column Table Mode, Instron Corp. Canton, Norwood, MA, USA). The crosshead moved at a constant speed of 0.33 mm/s. From the resulting force–time curve, the hardness (the maximum peak force during the first compression) was determined, equipped with 500 N load cell, Kramer S543A cutting cell, Drip Tray Food S5400A, and frame Food Support Frame S4427A. The test consisted of placing the cookie on the support, so that it was penetrated and compressed; in this way the cookies’ breakage or fracture resistance was determined [45].

#### 2.2.5. Sensory Analysis

##### Discriminative Test—Sorting

We worked with 102 consumers, aged between 18 and 30. Each consumer was given a cookie, on disposable plates coded with three random digits, randomly for each consumer. The test was carried out in the laboratory of the National University of Juliaca. The sorting test procedure was carried out in a single session divided into two stages according to what was established [46,47]. In the first stage, consumers tried each cookie, with the aim of forming groups of cookies according to their similarities or dissimilarities based on their overall sensory perception. In the second stage, each consumer assigned words (sensory attributes) to characterize the groups of cookies previously formed according to their perception [48].

##### Acceptability Test

The gluten-free cookie formulations were evaluated by the degree of consumer satisfaction based on the characteristics of smell, color, flavor, and general acceptance, using a nine-point hedonic scale. The samples were presented individually to 102 consumers from the city of Juliaca who regularly consume cookies. Samples were presented on disposable plates labeled with three-digit random numbers [49,50]. In both sensory tests, consumers agreed to participate voluntarily by giving their informed consent.

#### 2.2.6. Statistical Design

A Box–Behnken (DBB) design was applied considering cañihua flour, whey, and potato starch as variables in the formulation process, and the other inputs (Table 1) varied according to the design applied, obtaining a total of twelve treatments and three central points (Table 2). Through the experimental design, we sought to determine the influence of the factors of cañihua flour, whey, and potato starch. Furthermore, each formulation is a unique experimental condition, where each variation in cookie ingredients was carefully designed to explore its impact on different aspects of the cookies’ properties.

#### 2.2.7. Statistical Analysis

The statistical analysis of the experimental design data was carried out with the Statistic 13.0 program (trials version) in order to identify the significance of the factors (*p* < 0.05), and after that, contour graphs of the significant factors were created. For the analysis of sensory data for acceptability, a completely randomized block design and a comparison of means were applied using the 95% Tukey test, and for the discriminative sorting task test, a multivariate factor analysis was used. The data obtained were statistically treated with the statistical program R version 4.1.1.0.

## 3. Results and Discussion

### 3.1. Physicochemical and Proximal Analysis

To identify the nutritional contribution of the ingredients in the gluten-free cookies, the centesimal composition of the cañihua flour and of the potato starch was quantified: Moisture 9.00 ± 0.1 and 7.69 ± 0.3%, carbohydrates 69.59 ± 0.18 and 91.54 ± 0.34%, fat 3.61 ± 0.13 and 0.020 ± 0.01%, proteins 14.80 ± 0.2 and 0.570 ± 0.1%, and ashes 3.40 ± 0.25 and 0.150 ± 0.05%, respectively; the sweet whey had pH 5.80 ± 0.15, titratable acidity expressed in lactic acid was 0.110 ± 0.02%, fat was 0.30 ± 0.09%, proteins were 1.20 ± 0.03%, non-fat solids were 7.44 ± 0.04%, salts were 0.610 ± 0.01%, and lactose was 4.09 ± 0.01%.

**Figure 1:**
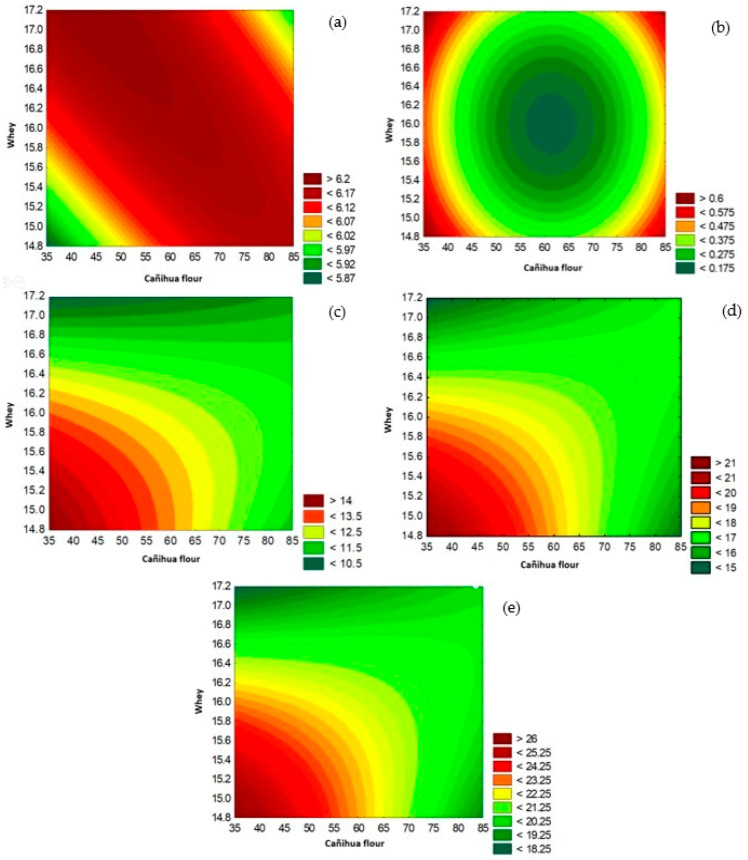
Influence of cañihua flour and whey on pH (**a**), acidity (**b**), a* (**c**), b* (**d**) and C* (**e**).

The moisture content, ash, pH, acidity, and water activity (physicochemical properties) of the gluten-free cookies are shown in Table 3. Significant differences between the proposed formulations, where formulations 2 and 15 have a greater percentage of moisture, is probably due to the starch content, which retains water on its surface [24]. Meanwhile, the pH ranges from 5.94 to 6.27 units, and the acidity (expressed in lactic acid) from 0.150 to 0.492%, a result that can be influenced by the addition of cañihua flour and whey in the formulation of the cookies that had a pH value of 5.80 units. According to the data shown in Table 3 with respect to the DBB model, the addition of starch, cañihua, and whey does not affect the variables of moisture, ash, and water activity for optimization purposes, but it does influence acidity and pH. Figure 1 shows the contour surface of the DBB, for cañihua flour and whey on pH (a), acidity (b), a* (c), b* (d), and C* (e), observing the effects of whey and cañihua flour on the response variable, which present a minimum acidity value for each substitution value in the formulation, which implies finding appropriate parameters so that they do not exceed the maximum acidity limit according to the regulations. In addition, a linear effect was presented in the pH, demonstrating the interaction between cañihua flour and whey; however, a quadratic effect was presented with respect to acidity. Likewise, the formulation with the lowest acidity content (F12) was the one with the greatest acceptability by consumers; it ranged from 0.150 ± 0.05% acidity, expressed in lactic acid, and a pH of 6.27 ± 0.08, presenting slight acidity; value that is within that reported in research that indicates a pH 5.80 to 6.51 [51]. These results are similar to those reported in cañihua bread with the addition of whey that presented a pH of 6.10 [14].

### 3.2. Texture Analysis

**Figure 2:**
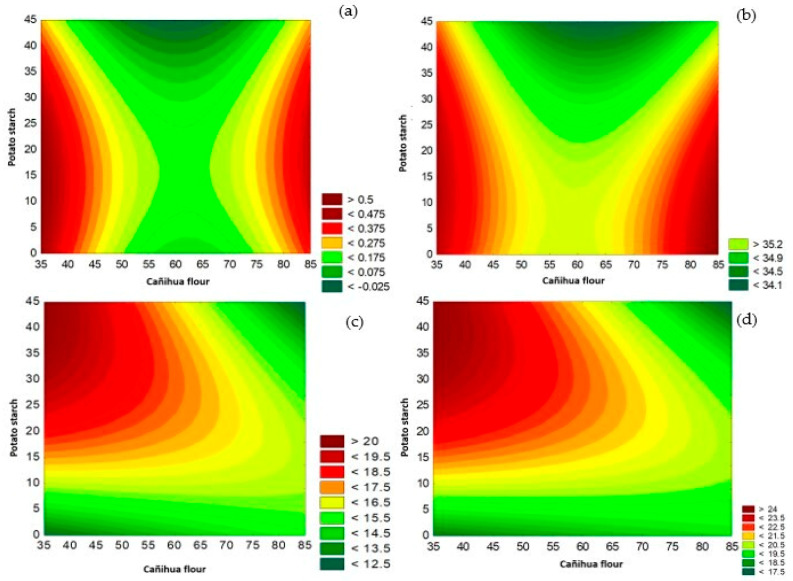
Influence of cañihua flour and potato starch on acidity (**a**), texture (**b**), L* (**c**), and C* (**d**).

Table 4 shows recorded values of the texture of gluten-free cookies; this parameter may represent a factor of acceptance or rejection by consumers. Of the proposed treatments, formulation F12 (32.53 ± 0.88 N) has lower hardness compared to the other formulations. These results show that hardness increases as the percentage of cañihua flour increases; in the same way, the starch content influences the texture, which can be attributed to the interaction of starch-proteins due to hydrogen bonds. Figure 2 shows the contour surface of the DBB for the cañihua flour and potato starch on the texture, indicating that the second-degree polynomial shows a better fit for the variables related to the texture when cañihua flour was increased in the formulation.

Texture is a critical aspect in gluten-free products [9] due to sensory perception, which is influenced by moisture content, with harder products requiring more extensive chewing [52]. The texture of the different formulations varied: formulation F1 (37.06 ± 0.59 N) presented a higher hardness value compared to F12 (32.53 ± 0.88 N), which was the formulation that had greater acceptability to consumers; this was due to the proportion of cañihua flour, potato starch, and whey in the formulation, which provides the variation in the moisture of the dough [52], meaning that the hardness ranged from 32.53 ± 0.88 N to 37.06 ± 0.59 N, with the values of hardness within those reported for cookies; thus, the carbohydrate content influences the variability of the texture of the product. On the other hand, the thickness of the cookies significantly influenced the variation in the texture parameters, and these variations are explained from the changes in the product after the thermal baking treatment, such as the gelatinization of starches, the denaturation of proteins, and the reduction of moisture [53].

### 3.3. Color Properties

Table 4 presents color values for the different cookie formulations, color being one of the most important quality properties for acceptability that influences consumer perception. The formulation with the highest acceptability, F12, presented 47.10 ± 0.65 CIELAB units of clarity (L*), which is reduced when the percentage of cañihua flour in the formulation increased. Likewise, the chromatic a* value presented a positive value of 9.78 ± 0.98 and a b* value of 15.44 ± 0.89, the chroma being the value that indicates the intensity of the color, decreasing when the formulation contained a higher content of cañihua flour and increasing when there was a higher content of potato starch in the formulation. Figure 2 shows the contour surface of the DBB for whey and potato starch on acidity, L*, a*, b*, and C*.

**Figure 3:**
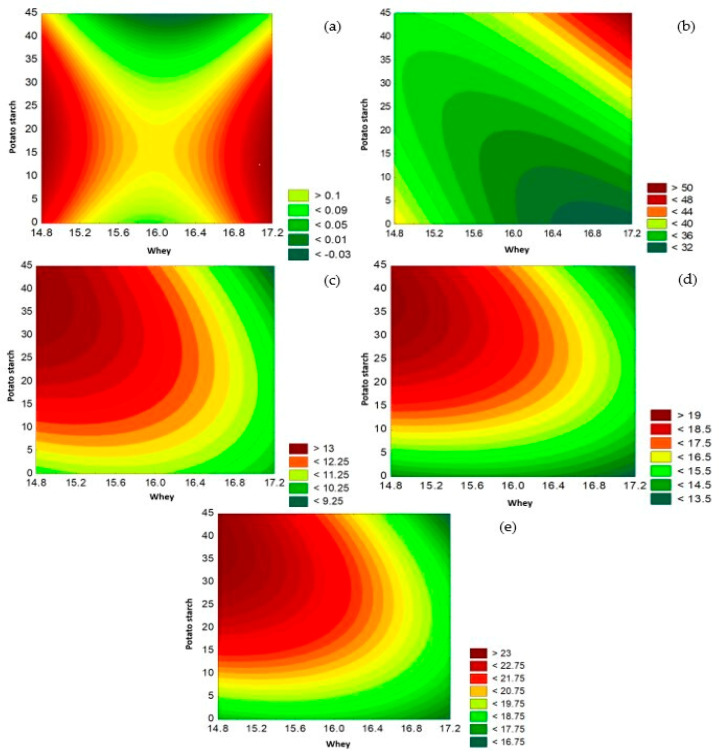
Influence of whey and potato starch on acidity (**a**), L* (**b**), a* (**c**), b* (**d**), and C* (**e**).

Therefore, the chromatic parameters shown in Table 4 show values of L*, a*, b* h*, and C*, which demonstrates that the values are high and positive due to the increase in cañihua flour in the formulation, finding the same relationship as [54], which mentions that the cookies have lower surface luminosity (L*) in the treatments that contain a lower cassava starch content and observes the tendency of cassava starch to decrease the color values a* and b*. On the other hand, the hue value (H*) increased with a higher content of whey and potato starch in the formulation, with a tendency to decrease as the cañihua flour increased, while the chroma value decreased as the flour increased. of cañihua and whey to the formulation, while its value increases with the addition of potato starch to at least 40 g, and above this there is a tendency to decrease, indicating that the chromatic parameter is lost due to the incorporation of cañihua flour into the formulation, which is why it has an impact on the color of the cookies and why the cookies differ in color [55]; however, the darker appearance of the cookies may be related to the Maillard reaction or caramelization that takes place during baking [56]. The results analyzed using the DBB show that the quadratic model fits better with respect to the chromatic parameters (a*, b*, C*, and L*). In the same way, the significance of the models was evaluated by analysis of variance, which presented a significant effect (*p* < 0.05). Figure 3 shows the contours generated to assess the significant influence of whey and potato starch on the acidity, L*, a*, b*, and C* parameters.

### 3.4. Sensory Analysis

#### 3.4.1. Sorting Task

**Figure 4:**
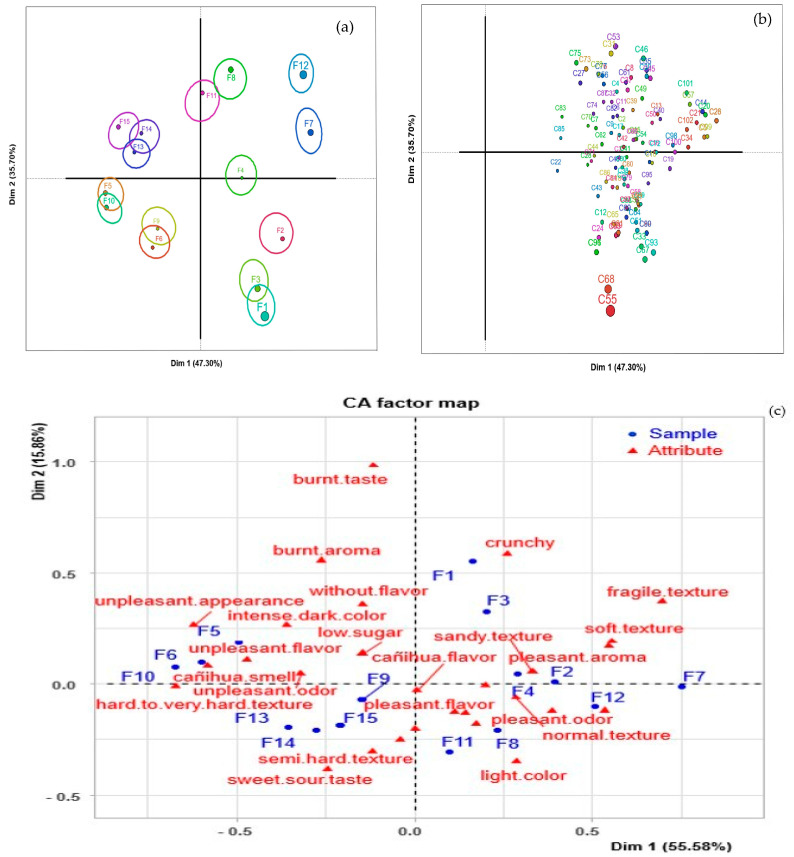
Sensory map of cookies (**a**); consumer (**b**) and correspondence analysis (**c**) using the sorting task test.

The sensory evaluation was carried out with 102 consumers who regularly consume cookies. Figure 4 shows the proportion of cookie samples which consumers and correspondence analysis agreed upon during the sorting task. Figure 4a shows the groupings of the samples made by consumers: the formation of nine groups is observed, the first group being F1 and F3, the second group F6 and F9, the third group F10 and F5, the fourth group F11 and F8, and the fifth F13, F14, and F15 (same formulation), indicating that participants perceived the repetitions in a similar way. The sixth, seventh, eighth and ninth groups independently comprised F2, F4, F7, and F12, respectively. The different groupings of the cookie samples present a clear separation between groups. Figure 4b shows the representation of consumers; an expected heterogeneity can be seen in the grouping of the samples, explained by the inter-individual variability between consumers. The introduction of heterogeneity in choice models, market segmentation, positioning, and micro-marketing allows us to see a group of consumers with the same tendencies or choices, because consumers are diverse and expect their individuality to be recognized [57]. 

Figure 4c presents the sensory map through CA, explaining 71.44% of the total variability in the first two dimensions. The location of the samples was made based on the attributes described by consumers, and six groups were formed, the first group (F1 and F3) described as crunchy, the second group (F2, F4, and F12) characterized by a sandy texture, normal texture, and pleasant odor. The third group (F7) was recognized by a soft and fragile texture, the fourth group (F11 and F8) by a light color, the fifth group (F9, F13, F14, and F15) by a semi-hard texture and sweet-sour taste, and finally the sixth group (F5, F6, and F10) by a Cañihua smell, unpleasant odor, unpleasant flavor, and unpleasant appearance. Sensorily, the samples of F9 are not expected to be described the same way as those of F13, F14, and F15 (the same product) due to the absence of potato starch; however, consumers did not perceive this absence and described it similarly to these samples. The sorting task method allows us to know the perception of consumers regarding the sensory characteristics of cookies, enabling the classification or grouping of the samples according to their similarities and/or dissimilarities.

#### 3.4.2. Acceptability

**Figure 5:**
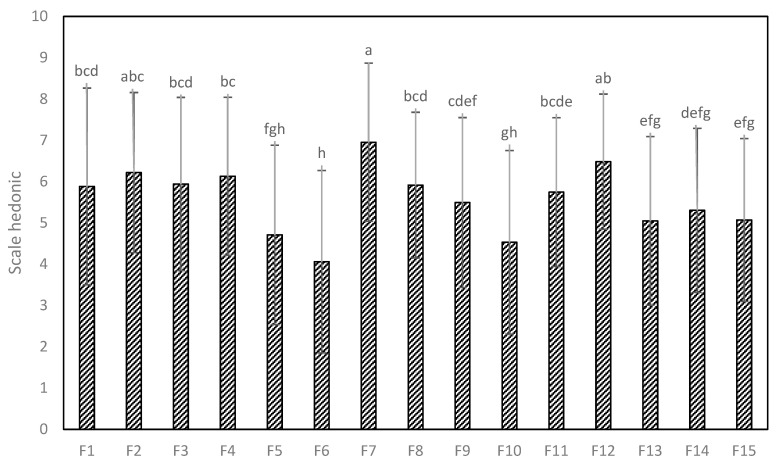
Results of the acceptability of the different samples evaluated by consumers (n = 102). ^a,b,c,d,e,f,g,h^ Different letters indicate significant differences (*p* < 0.05).

Figure 5 shows the results of consumer acceptability using a nine-point structured hedonic scale. Significant differences were found between the samples studied (*p* < 0.05). The samples of F7, F2, and F12 had greater acceptability (I like it a lot); these cookies did not present significant differences (*p* > 0.05), that is, they were statistically similar. The least acceptability was presented for F5, F6, and F10 (neither like nor dislike). The rest of the samples were rated from like it slightly to like it moderately. These results are similar to those reported by Krajewska et al. [58], in cookies enriched with fruits and by-products, and Silva et al. [59], in gluten-free cookies. In general, cookies can be enriched with Cañihua flour, whey, and potato starch without a significant impact on the consumer’s perception of the products. However, if a higher level of supplementation is desired (<80%), further experiments need to be performed to improve the sensory characteristics of the final cookies.

### 3.5. Evaluation of the Formulation with the Best Physicochemical and Sensory Characteristics

The product with the best physicochemical and sensory characteristics was found to be the F12 formulation, which presented values of moisture 4.83 ± 0.05%, protein 7.15 ± 0.87%, fat 15.09 ± 0.15%, crude fiber 1.05 ± 0.05%, ash 2.70 ± 0.08%, carbohydrates 73.51%, and 458.45 kcal/100 g. Likewise, the high protein content of the cookie with the best acceptability could be attributed to the proteins from cañihua flour and whey. Whey protein is effective for protein synthesis in muscles, demonstrating functionality due to its content in bioactive components derived from whey, such as essential amino acids, micronutrients, β-Lactoglobulin, α-Lactalbumin, Immunoglobulins, Bovine serum albumin, Guanosine monophosphate, Lactoferrin, and Lipopolysaccharide, which have many biomedical, pharmaceutical, and therapeutic applications, such as the prevention of type 2 diabetes, obesity, cardiovascular diseases, phenylketonuria, the elimination of excess free radicals produced by oxidative stress, the suppression of tumor development, anti-proliferative effects, and the treatment of metastatic carcinoma [60]. Thus, its use in the formulation allows for improvements in the technological properties of the dough to produce gluten-free functional foods [61].

In general, cañihua flour is characterized by its excellent nutritional profile, being a rich source of protein compared to similar raw materials such as quinoa, which contains 14.1% [62]; this is why there is growing interest in its use for the formulation of healthy food products with better nutritional value [63], allowing for an improvement in the nutritional quality of cookies [64,65] and being an alternative to meet the demand for gluten-free bakery products by groups with celiac disease, gluten sensitivity, and/or health awareness [66].

## 4. Conclusions

Gluten-free cookies were developed with Cañihua flour, whey, and potato starch. The incorporation of these ingredients produces significant differences in the physicochemical, nutritional, colorimetric, and texture parameters. The F12 formulation presented better physicochemical characteristics and sensory attributes. This formulation contains 38.51% Cañihua flour, 10.91% whey, 25.69% potato starch, 8.34% margarine, 11.10% sugar, 0.19% sodium chloride, 0.51% baking powder, 0.51% vanilla essence, and 4.24% egg, as well as containing 0% gluten and showing a good protein profile of 7.75%; it also met all the physicochemical and nutritional quality parameters, so the results of this research demonstrate that it is possible to formulate cookies using flour from high Andean crops such as cañihua flour, which is an adequate and acceptable resource for celiac consumers and can be used for the production of cookies. In addition, the production of such cookies would take advantage of the whey produced and discarded by dairy plants.

## Figures and Tables

**Table 1:** Formulation of the different cookies prepared (%).

Ingredients	F1	F2	F3	F4	F5	F6	F7	F8	F9	F10	F11	F12	F13-F14-F15 *
Cañihua flour	35.15	52.02	34.54	51.35	42.19	59.35	29.67	45.77	52.72	51.81	39.01	38.51	44.51
Whey	13.18	9.75	14.68	10.91	16.88	11.87	11.87	9.15	13.18	14.68	9.75	10.91	11.87
Potato starch	17.59	13.00	17.27	12.85	0.00	0.00	29.69	22.87	0.00	0.00	26.01	25.69	14.85
Margarine	11.42	8.45	11.23	8.34	13.72	9.65	9.64	7.44	11.44	11.23	8.45	8.34	9.64
Sugar	15.20	11.25	14.94	11.10	18.25	12.83	12.83	9.90	15.20	14.94	11.25	11.10	12.83
Sodium chloride	0.26	0.20	0.26	0.19	0.32	0.22	0.22	0.17	0.26	0.26	0.20	0.19	0.22
Baking powder	0.70	0.52	0.69	0.51	0.84	0.59	0.59	0.46	0.70	0.69	0.52	0.51	0.59
Vanilla essence	0.70	0.52	0.69	0.51	0.84	0.59	0.59	0.46	0.70	0.69	0.52	0.51	0.59
Egg	5.80	4.29	5.70	4.24	6.96	4.90	4.90	3.78	5.80	5.70	4.29	4.24	4.90

* F13, F14 and F15 have the same formulation.

**Table 2:** Formulation of gluten-free cookies for celiac using a Box–Behnken design.

Formulations	Coded Variables			Decoded Variables (g)		
	X1	X2	X3	HC *	L *	AP *
F1	−1	−1	0	40	15	20
F2	1	−1	0	80	15	20
F3	−1	1	0	40	17	20
F4	1	1	0	80	17	20
F5	−1	0	−1	40	16	0
F6	1	0	−1	80	16	0
F7	−1	0	1	40	16	40
F8	1	0	1	80	16	40
F9	0	−1	−1	60	15	0
F10	0	1	−1	60	17	0
F11	0	−1	1	60	15	40
F12	0	1	1	60	17	40
F13	0	0	0	60	16	20
F14	0	0	0	60	16	20
F15	0	0	0	60	16	20

* HC = Cañihua flour; L = Whey and AP = Potato starch.

**Table 3:** Analysis of moisture, ash, pH, acidity, Aw of the cañihua cookie treatments.

Formulation	Moisture (%)	Ash (%)	pH	Acidity (%)	Aw
F1	4.52 ± 0.02	3.61 ± 0.03	5.94 ± 0.03	0.492 ± 0.08	0.410 ± 0.04
F2	5.28 ± 0.01	2.60 ± 0.04	6.20 ± 0.04	0.486 ± 0.09	0.410 ± 0.03
F3	3.12 ± 0.02	2.89 ± 0.09	6.18 ± 0.05	0.468 ± 0.08	0.400 ± 0.04
F4	4.54 ± 0.01	2.63 ± 0.08	6.08 ± 0.06	0.468 ± 0.05	0.400 ± 0.05
F5	4.82 ± 0.03	2.66 ± 0.07	6.14 ± 0.04	0.378 ± 0.06	0.390 ± 0.04
F6	4.20 ± 0.02	2.83 ± 0.03	6.01 ± 0.12	0.234 ± 0.04	0.400 ± 0.03
F7	5.13 ± 0.04	3.02 ± 0.04	6.19 ± 0.10	0.246 ± 0.08	0.410 ± 0.04
F8	4.32 ± 0.08	2.76 ± 0.04	6.27 ± 0.09	0.186 ± 0.09	0.420 ± 0.04
F9	4.64 ± 0.02	3.19 ± 0.03	6.11 ± 0.08	0.192 ± 0.08	0.405 ± 0.05
F10	4.39 ± 0.03	3.08 ± 0.07	6.12 ± 0.09	0.252 ± 0.07	0.410 ± 0.03
F11	3.75 ± 0.04	2.26 ± 0.06	6.19 ± 0.09	0.180 ± 0.04	0.400 ± 0.02
F12	4.83 ± 0.05	2.70 ± 0.08	6.27 ± 0.08	0.150 ± 0.05	0.400 ± 0.05
F13	3.83 ± 0.06	2.76 ± 0.09	6.14 ± 0.10	0.162 ± 0.06	0.410 ± 0.03
F14	4.59 ± 0.05	3.05 ± 0.02	6.19 ± 0.09	0.162 ± 0.04	0.400 ± 0.02
F15	5.42 ± 0.07	3.08 ± 0.08	6.26 ± 0.08	0.162 ± 0.03	0.410 ± 0.03
Regression coefficients					
HC	−0.118	−0.150	0.090	−0.060	0.010
HC^2^	−0.001	−0.010	−0.010	0.010	−0.010
L	6.551	−0.090	1.510	−3.970	−0.020
L^2^	−0.236	−0.020	−0.040	0.130	0.010
AP	−0.265	−0.090	−0.020	0.020	−0.020
AP^2^	0.010	−0.020	0.010	−0.010	0.010
HC*L	0.080	0.090	−0.050	0.001	−0.000
HC*AP	−0.010	−0.010	0.010	0.001	−0.000
L*AP	0.020	0.070	0.010	−0.001	0.010
*p*-value					
HC	0.782	0.190	0.042 *	0.031 *	0.587
HC^2^	0.969	0.543	0.085	0.001 *	0.756
L	0.622	0.153	0.491	0.047 *	0.424
L^2^	0.638	0.307	0.218	0.001 *	0.950
AP	0.999	0.951	0.215	0.070	0.783
AP^2^	0.974	0.759	0.625	0.004 *	0.950
HC*L	0.732	0.317	0.018 *	0.935	1.000
HC*AP	0.922	0.985	0.101	0.283	0.009
L*AP	0.489	0.336	0.533	0.254	0.698

* The variable significantly influences (*p* < 0.05).

**Table 4:** Texture analysis and chromatic parameters of the cañihua cookie treatments.

Formulation	Texture (N)	L*	a*	b*	h*	C*
F1	37.06 ± 0.59	40.52 ± 0.89	14.33 ± 0.56	20.63 ± 0.67	0.960 ± 0.17	25.12 ± 0.97
F2	35.77 ± 0.69	36.72 ± 0.91	11.51 ± 0.58	16.04 ± 0.78	0.950 ± 0.26	19.75 ± 0.92
F3	35.97 ± 0.88	38.12 ± 0.89	11.25 ± 0.86	15.83 ± 0.65	0.950 ± 0.10	19.42 ± 0.34
F4	35.97 ± 1.08	33.34 ± 0.72	11.29 ± 0.57	16.84 ± 0.43	0.980 ± 0.21	20.27 ± 0.45
F5	36.26 ± 1.18	33.54 ± 0.96	10.82 ± 0.78	14.21 ± 0.56	0.920 ± 0.13	17.86 ± 0.53
F6	36.75 ± 0.88	33.93 ± 0.76	10.72 ± 0.89	15.82 ± 0.45	0.970 ± 0.16	19.11 ± 0.43
F7	35.97 ± 0.69	43.26 ± 0.64	13.59 ± 0.83	20.16 ± 0.67	0.980 ± 0.18	24.31 ± 0.65
F8	35.47 ± 0.59	36.76 ± 0.89	11.79 ± 0.54	15.21 ± 0.97	0.910 ± 0.12	19.24 ± 0.64
F9	35.18 ± 0.39	39.36 ± 0.87	11.34 ± 0.73	15.04 ± 0.87	0.920 ± 0.08	18.84 ± 0.98
F10	35.28 ± 0.69	34.40 ± 0.67	10.44 ± 0.89	14.36 ± 0.56	0.940 ± 0.07	17.75 ± 0.78
F11	34.88 ± 0.59	35.23 ± 0.86	13.52 ± 0.87	19.52 ± 0.65	0.970 ± 0.02	23.74 ± 0.89
F12	32.53 ± 0.88	47.10 ± 0.65	9.78 ± 0.98	15.44 ± 0.89	1.000 ± 0.21	18.28 ± 0.67
F13	35.08 ± 0.78	36.06 ± 0.85	12.50 ± 0.76	16.93 ± 0.76	0.930 ± 0.12	21.05 ± 0.89
F14	35.77 ± 0.68	35.84 ± 0.76	12.96 ± 0.89	18.21 ± 0.87	0.950 ± 0.09	22.36 ± 0.76
F15	33.01 ± 0.88	33.01 ± 0.93	11.97 ± 0.67	18.3 ± 0.89	0.990 ± 0.07	21.87 ± 0.95
Regression coefficients						
HC	−0.369	0.182	−0.590	−1.047	−0.005	−1.197
HC^2^	0.003	0.001	0.001	−0.001	−0.001	−0.000
L	18.415	−73.070	11.089	7.405	−0.271	12.527
L^2^	−0.572	2.179	−0.422	−0.370	0.007	−0.548
AP	0.538	−3.162	0.742	1.129	0.001	1.343
AP^2^	−0.001	0.004	−0.002	−0.003	−0.001	−0.003
HC*L	0.004	−0.012	0.035	0.069	0.001	0.078
HC*AP	−0.001	−0.004	−0.001	−0.001	−0.000	−0.003
L*AP	−0.031	0.210	−0.035	−0.040	0.000	−0.054
*p*-value						
HC	0.841	0.082	0.180	0.002 *	0.337	0.002 *
HC^2^	0.009 *	0.984	0.900	0.688	0.786	0.804
L	0.182	0.874	0.290	0.054	0.250	0.797
L^2^	0.076	0.141	0.198	0.215	0.602	0.097
AP	0.021 *	0.026 *	0.004 *	0.001 *	0.836	0.010 *
AP^2^	0.179	0.193	0.040 *	0.003 *	0.603	0.001 *
HC*L	0.777	0.846	0.048 *	0.003 *	0.456	0.002 *
HC*AP	0.365	0.210	0.181	0.001 *	0.069	0.002 *
L*AP	0.055	0.017 *	0.049 *	0.019 *	0.676	0.008 *

* The variable significantly influences (*p* < 0.05).

## Data Availability

The original contributions presented in the study are included in the article, further inquiries can be directed to the corresponding author.
